# Dysregulated expression of IDO may cause unexplained recurrent spontaneous abortion through suppression of trophoblast cell proliferation and migration

**DOI:** 10.1038/srep19916

**Published:** 2016-01-27

**Authors:** Shanshan Zong, Chunqing Li, Chengfeng Luo, Xin Zhao, Chunhong Liu, Kai Wang, Wenwen Jia, Mingliang Bai, Minghong Yin, Shihua Bao, Jie Guo, jiuhong Kang, Tao Duan, Qian Zhou

**Affiliations:** 1 Clinical and Translational Research Center, Shanghai First Maternity and Infant Hospital, Tongji University School of Medicine, Shanghai, 200040, China; 2 Clinical and Translational Research Center, Shanghai First Maternity and Infant Hospital Shanghai Key Laboratory of Signaling and Disease Research, School of Life Science and Technology, Tongji University, Shanghai 200092, China; 3 Department of Gynaecology, Shanghai First Maternity and Infant Hospital, Tongji University School of Medicine, Shanghai, 200040, China; 4 Department of Obstetrics and Gynaecology, Punan hospital, Shanghai, 2000125, China; 5 Department of Obstetrics, Shanghai First Maternity and Infant Hospital, Tongji University School of Medicine, Shanghai, 200040, China

## Abstract

In pregnancy, trophoblast proliferation, migration and invasion are important for the establishment and maintenance of a successful pregnancy. Impaired trophoblast function has been implicated in recurrent spontaneous abortion (RSA), a major complication of pregnancy, but the underlying mechanisms remain unclear. Indoleamine 2,3-dioxygenase (IDO), an enzyme that catabolizes tryptophan along the kynurenine pathway, is highly expressed in the placenta and serum during pregnancy. Here, we identified a novel function of IDO in regulating trophoblast cell proliferation and migration. We showed that IDO expression and activity were decreased in unexplained recurrent spontaneous abortion (URSA) compared to normal pregnancy. Furthermore, blocking IDO in human trophoblast cells led to reduced proliferation and migration, along with decreased STAT3 phosphorylation and MMP9 expression. Increased STAT3 phosphorylation reversed the IDO knockdown-suppressed trophoblast cell proliferation and migration. In addition, the overexpression of IDO promoted cell proliferation and migration, which could be abolished by the STAT3 signaling inhibitor (AG490). Finally, we observed similar reductions of STAT3 phosphorylation and MMP9 expression in URSA patients. These results indicate that the level of IDO expression may be associated with pregnancy-related complications, such as URSA, by affecting trophoblast cell proliferation and migration via the STAT3 signaling pathway.

Recurrent spontaneous abortion (RSA), defined by at least two consecutive losses of pregnancy before 20 weeks gestation with the same partner, affects approximately 1–5% of reproductive-age women around the world^1^,^2^. The etiology of RSA is still not well understood. Although some causes including infection, chromosomal abnormality, anatomic deformation, endocrine, metabolic, and autoimmune diseases, approximately 50% of cases have no known cause and are referred to as unexplained recurrent spontaneous abortion (URSA)^3^. Trophoblast cells are the most important cells in early pregnancy and are essential to both placental and fetal development. Defects in trophoblast cell function resulted in impaired uterine spiral artery rebuilding and have been implicated in pregnancy-related complications such as RSA, intrauterine growth retardation, and pre-eclampsia^4^,^5^.

Indoleamine 2,3-dioxygenase (IDO) is a cytoplasmic enzyme that degrades the essential amino acid tryptophan into kynurenine and kynurenic acid via the kynurenine pathway. During pregnancy, there were high levels of IDO expression in the placenta and serum, and IDO expression was reduced to the non-pregnancy level after delivery^6^. It has been known for decades that IDO is highly expressed in the placenta, but its physiological role in normal human pregnancy and its pathology, especially in relation to URSA, have not been well investigated.

IDO has been shown to be important in maintaining maternal-fetal tolerance. The use of IDO inhibitor could result in abortion in pregnant mice. After IDO blockage, an inflammatory reaction was observed in the maternal-fetal interface^7^,^8^,^9^. IDO expression at the maternal-fetal interface is lower in URSA than in normal early pregnancy. This reduction in URSA patients was observed at both the protein and mRNA levels in the placenta and decidua^10^. In one report, 30% of spontaneous miscarriage patients had a reduction in the proportion of IDO-positive cells within the decidua^11^. Another report showed that IDO positive monocytes cells and dendritic cells from the peripheral blood were reduced in spontaneous abortion^12^.

Trophoblast cells are like tumor cells in that proliferation and invasion are common features, and both express high levels of IDO. Recent evidences have shown that IDO facilitates proliferation and metastasis in several types of tumors^13^,^14^,^15^,^16^. Whether IDO regulates trophoblast cell function has not yet been carefully investigated. Therefore, we studied the role of IDO in trophoblast cell proliferation and migration in URSA.

In this study, we compared IDO expression and activity in placental villi between normal early pregnancy and URSA. Using the MTS cell proliferation assay and transwell migration assay, manipulating IDO activity in human trophoblast cell lines by both genetic and pharmacologic approaches, we elucidated the role of IDO in trophoblast cell proliferation and migration. Results showed that the expression of IDO is lower in URSA patients than in normal pregnant women. Western blot analysis revealed that IDO knockdown inhibits cell proliferation and migration followed by a decrease in STAT3 phosphorylation. In addition, the overexpression of IDO promotes cell proliferation and migration, which could be abolished by AG490. Furthermore, we found that STAT3 phosphorylation in URSA patients was lower compared to normal pregnant women.

## Materials and Methods

### Clinical samples

Villus tissues were obtained from 40 women undergoing voluntary medical abortion in the outpatient operating room of the Department of Gynecology at Shanghai First Maternity and Infant Hospital (Tongji University, Shanghai, China) from May 2013 to March 2014. The URSA group comprised 20 women with a history of two to six spontaneous miscarriages at early pregnancy (7–10 weeks gestation) who had not previously been investigated. Their average number of abortions was 3.15 ± 1.15, and their mean age was 30.5 ± 0.96 years. URSA was diagnosed after excluding any verifiable causes, including infection, endocrine or metabolic disease, chromosomal abnormality, anatomic deformation, and autoimmune response. All patients had a single male partner for the pregnancies under investigation. All patients and their male partners had normal karyotypes^17^, and all male partners had a normal semen status according to criteria from the World Health Organization^18^.

The control group comprised 20 randomly selected women who underwent a legal termination of an apparently normal early pregnancy (7–10 weeks gestation) at the same facility during the same period. The mean age of the control group was 31.3 ± 0.94 years, and there was no significant difference in age or gestation between the URSA and control groups. All control subjects had one or two living children and no history of spontaneous abortion, ectopic pregnancy, preterm delivery or stillbirth. The average number of deliveries was 1.36 ± 0.48. In all cases, fetal heart activity was verified within 2 weeks of abortion and the embryonic karyotype was normal.

The majority of each sample was immediately frozen and stored in liquid nitrogen for mRNA and protein measurement. The remaining portion of each sample was immediately fixed for 24 h in cold 4% paraformaldehyde and embedded in paraffin wax for immunohistochemistry.

All procedures for sample collection and utilization were approved by the Shanghai First Maternity and Infant Hospital Scientific and Ethics Committees, and written informed consent was obtained from each participant prior to sample collection. The methods were carried out in accordance with the approved guidelines.

### Cell culture and infection

HTR-8/SVneo cells were cultured in DMEM/F12 medium (GIBCO, Grand Island, NY) supplemented with 10% FBS, 100 U/ml penicillin, and 100 mg/ml streptomycin (GIBCO) at 37 °C under 5% CO_2_ humidified air according to standard procedures.

IDO gene knockdown was performed using a short hairpin RNA (shRNA) lentiviral vector (Zhongqiaoxinzhou Biological Technology, Shanghai, China) according to the manufacturer’s protocol. Briefly, HTR-8/SVneo cells were seeded at 2 × 10^5^ cells/well in a 6-well plate. After adherent cells reached ~40% confluence, they were infected with an IDO-specific shRNA virus (shIDO1 or shIDO2) or a control shRNA (shCTL) supplemented with 8 μg/ml polybrene (Sigma, St. Louis, MO). Treated cells were selected with puromycin to generate puromycin-resistant clones, which were assayed by real-time PCR and western blotting.

For gene overexpression, the recombinant lentiviruses carrying IDO or STAT3 or control (Zhongqiaoxinzhou Biological Technology) were used according to the manufacturer’s protocol. The procedures were carried out as described above. Stably infected cells were selected and processed for further analysis by western blotting.

### Immunohistochemistry

Four-millimeter thick sections were cut from the paraffin-embedded tissue blocks by a sledge microtome and mounted onto 3-amino-propyl-tri-ethoxy-silane (APES)-coated glass slides, deparaffinized with xylol and rehydrated. For immunohistochemical detection, tissue slides were deparaffinized and rehydrated. Endogenous peroxidase activity was blocked with 3% hydrogen peroxide/methyl alcohol for 10 min. Nonspecific binding sites were blocked with 5% BSA for 30 min. Slides were incubated with primary monoclonal anti-IDO (1:50, Millipore, Billerica, MA) at 4 °C for 12 h and then incubated for 30 min with secondary anti-mouse antibody conjugated with horseradish peroxidase and exposed to DAB (Gene Technology Shanghai Company, Shanghai, China). As a negative control, the primary antibody was replaced with rabbit preimmune IgG (1:2000, Kangchen Bio, Shanghai, China).

### RNA isolation and real-time PCR

Total RNA was extracted using Trizol reagent (TaKaRa Bio, Dalian, China) and RNAsimple Total RNA kit (Tiangen Biotech, Beijing, China) according to the manufacturer’s instructions. All RNA samples were treated with Turbo DNase enzyme (Applied Biosystems). RNA was quantified by UV absorption, and 1 μg total RNA was reverse-transcribed using the PrimeScript RT Reagent kit (TaKaRa) to make cDNA. Real-Time PCR was carried out using SYBR Premix Ex Taq (TaKaRa) according to the manufacturer’s instructions using primer sequences from Sangon (Shanghai, China). Primers were as follows: IDO-Forward: 5′-GGCTTCTTCCTCGTCTCTCTATTG-3′; IDO-Reverse: 5′-TGACGCTCTACTGCACTGGATAC-3′; GAPDH-Forward: 5′-ACTCCACGACGTACTCAGCG-3′; GAPDH-Reverse: 5′-GGTCGGACTCAACGGATTTG-3′. Reactions were performed with the Applied Biosystems 7500 Fast Real-time PCR System (Life Technologies, Carlsbad, CA). Levels of mRNA expression were analyzed using the 2^−ΔΔCT^ method and normalized to GAPDH as the housekeeping gene control.

### Western blotting

Tissues or cells were lysed, supernatants were collected and protein concentration was determined with the BCA Protein Assay Reagent (Thermo Scientific, Waltham, MA). Lysates (30 μg for cells, 80 μg for tissues) were separated by 10% SDS-PAGE and transferred electrophoretically onto polyvinylidene difluoride membranes. After blocked with TBST containing 5% non-fat milk, membranes were incubated with the following primary antibodies: anti-IDO (1:500, Millipore, Billerica, MA, No. MAB5412), anti-pSTAT3 (1:2000, Cell Signaling Technology, Beverly, MA, No. 4113), anti-STAT3 (1:2000, Cell Signaling Technology, No. 4904), anti-MMP9 (1:1000, Cell Signaling Technology, No. 3852), and anti-GAPDH (1:5000, Kangchen, No. KC-5G4). After incubation with an HRP-conjugated anti-mouse secondary antibody (1:2000, Cell Signaling Technology, No. 7076) or anti-rabbit secondary antibody (1:2000, Cell Signaling Technology, No. 7074), products were visualized with ECL reagents (Thermo Scientific) and immunoreactive signals were analyzed by densitometry. The intensity of each target protein band was quantified by densitometry analysis using Image J software (version 1.46r, National Institutes of Health, Bethesda, MD). GAPDH was used as an endogenous loading control.

### HPLC analysis of IDO activity

HPLC assays were performed by Zhangjiang Biological Technology (Shanghai, China). Frozen villus samples were homogenized in 2 μl perchloric acid (3.6%, Sigma, St. Louis, MO) per mg sample weight and microcentrifuged at 14,000 rpm for 20 min at 4 °C. The protein-free supernatant was passed through a 0.2-μm filter. Samples (20 μl) were injected by a multi-sampler onto a C-18 column. Elution and detection of tryptophan (Trp) and kynurenine (Kyn) were performed as described by Laich *et al*^19^. IDO activity was represented as the Kyn/Trp ratio measured by HPLC.

### Cell proliferation assays

Cells were seeded in 96-well plates at 2000 cells/well and cultured in 100 μl DMEM/F12 for 16 h. After adherence, cells were treated with different concentrations of the IDO inhibitor Levo-1-Methyl-Tryptophan (l-1mT, 250–4000 μM; Sigma), the STAT3 phosphorylation activator Leukemia Inhibitor Factor (LIF, 50 ng/ml, R&D, Minneapolis, MN), the STAT3 phosphorylation inhibitor AG490 (5–80 μM; Sigma) or vehicle control for 48 h. Next, 20 μl of the CellTiter 96**®** AQueous One Solution Cell Proliferation Assay (MTS, Promega, Beijing, China) was added to each well and the plates were incubated for 1 h. The cell numbers in each well were determined by optical density at 490 nm using a spectrophotometer. Standard curves were established with known cell numbers (625, 1250, 2500, 5000, 10000, 20000 and 40000 cells/well). For IDO knockdown without or with STAT3 overexpression proliferation assays, cells were seeded into 96-well plates at 2000 cells/well with 100 μl medium. Cells were incubated without any vehicle for 48 h and processed as described above. Three independent experiments were performed with six technical replicates for each sample.

We prepared 500 mM 1-MT using 0.1 M NaOH and adjusted the pH to 7.5 using 1 M HCl. The same volume of mixture of 0.1 M NaOH and 1 M HCl with pH 7.5 was used as a vehicle control for 1-l-MT. The 10 mM AG490 liquid in DMSO was purchased from Sigma, and the same volume of DMSO was used as a vehicle control.

### Cell migration assays

All assays were conducted using the Transwell system (24-Multiwell BD Falcon FluoroBlok Insert System, 8-μm pores). Cells were treated with vehicle control, l-1mT (0, 1000 or 2000 μM), LIF (50 ng/ml) or AG490 (20μM) for 48 h, then seeded at 50000 cells/well in medium containing 1% FBS in the upper chamber. Medium containing 10% FBS was added to the lower chamber. After 16 h treatment with vehicle control, l-1mT (0, 1000 or 2000 μM), LIF (50 ng/ml) or AG490 (20μM), cells that migrated to the bottom of the inserts were stained with calcein AM (0.2 μg/ml; Invitrogen, Carlsbad, CA) for 30 min and recorded using an inverted microscope mounted with a CCD camera. Migrated cells were examined and counted using Metamorph image analysis software (Molecular Devices, Sunnyvale, CA). For IDO knockdown with or without STAT3 overexpression migration assays, cells were seeded at 50000 cells/well in medium with 1% FBS in the upper chamber and incubated without any vehicle for 16 h, the assay was carried out as described. Three independent experiments were performed with two technical replicates for each sample. Representative images are shown, and data are expressed as the means ± SEM % of the controls.

### Statistical analysis

All statistical analyses were performed with Student’s t-test. A two-tailed P < 0.05 was considered statistically significant. All cell-based experiments were performed in quadruplicate and repeated three times.

## Results

### Expression and activity of IDO was lower in villi from the URSA group than the control group

**Figure 1:**
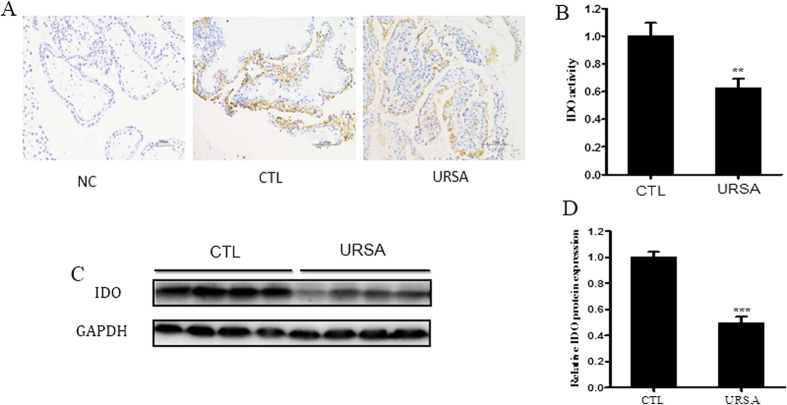
IDO expression and activity are decreased in villi from URSA patients compared to those from normal pregnant women (CTL). (**A**) Representative images of IDO expression in villous tissue. Immunostaining verifies IDO expression in tissue sections. A Brownish color represents positive staining. Magnification: 200×. (**B**) HPLC analysis of IDO activity, expressed as the ratio of Kyn to Trp. (**C**) Western blot analysis of IDO protein using GAPDH as an internal control. (**D**) Statistical analysis of western blot results in C (n = 20; Student’s t-test; *p < 0.05, ***p < 0.001).

We analyzed IDO protein expression and enzymatic activity in villi of women with normal pregnancy and URSA using immunohistochemistry (Fig. 1A), HPLC (Fig. 1B) and western blotting (Fig. 1C,D). Immunohistochemistry demonstrated that trophoblast cells expressed IDO protein. IDO protein level and activity were both decreased in the URSA group compared to the control group.

### IDO-specific shRNA knocked down IDO expression in HTR-8/SVneo cells

**Figure 2:**
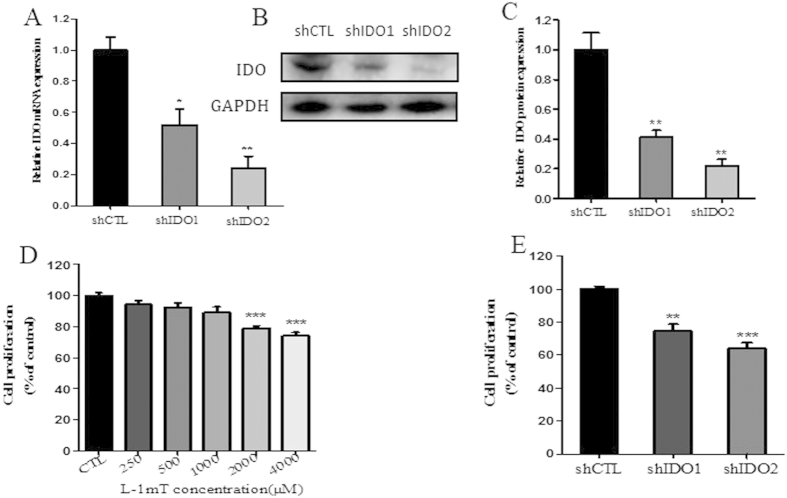
Inhibition of IDO with l-1mT or IDO-specific shRNA suppressed proliferation of HTR-8/SVneo cells. (**A**) Real-time PCR analysis of IDO mRNA in HTR-8/SVneo cells infected with IDO shRNA or control (CTL) shRNA (n = 3; Student’s t-test; *p < 0.05, **p < 0.01). (**B**) Western blot analysis of IDO protein in HTR-8/SVneo cells infected with IDO-specific or control shRNA. GAPDH was used as an internal control. (**C**) Statistical analysis of protein expression in B (n = 3; Student’s t-test; **p < 0.01). (**D**) Proliferation of HTR-8/SVneo cells treated with vehicle or l-1mT (250−4000 μM; n = 3; Student’s t-test; ***p < 0.001). (**E**) Proliferation of HTR-8/SVneo cells infected with control or IDO-specific shRNA (n = 3; Student’s t-test; **p < 0.01, ***p < 0.001).

The effect of IDO knockdown on mRNA and protein expression was verified by real-time PCR (Fig. 2A) and western blotting (Fig. 2B,C). IDO expression was significantly decreased in both knockdown cell lines (shIDO1 and shIDO2) compared to the control knockdown line (shCTL). shIDO2 knockdown cells showed more obvious reductions than shIDO1 knockdown cells.

### IDO inhibition and knockdown suppressed trophoblast cell proliferation and migration

**Figure 3:**
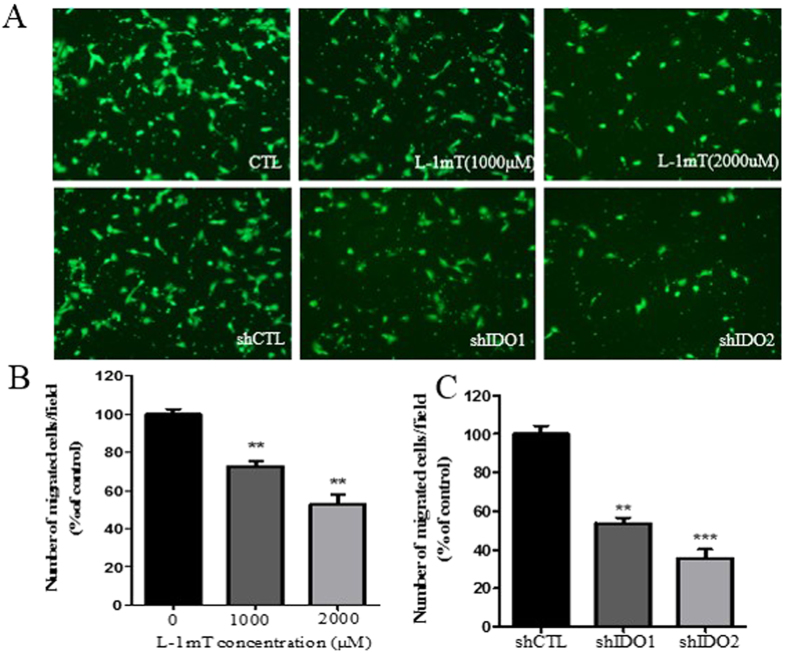
Inhibition of IDO with l-1mT or IDO-specific shRNA suppressed the migration of HTR-8/SVneo cells. (**A**) Representative images from Transwell migration assay. Cells were treated with vehicle or l-1mT (top) or with control or IDO-specific shRNA (bottom). (**B**) Statistical analysis of the effect of l-1mT on HTR-8/SVneo cell migration (n = 3; Student’s t-test; **p < 0.01). (**C**) Statistical analysis of the effect of IDO knockdown on HTR-8/SVneo cell migration (n = 3; Student’s t-test; **p < 0.01, ***p < 0.001).

To study the roles of IDO in trophoblast cell proliferation and migration, we used the IDO inhibitor l-1mT and shRNA in HTR-8/SVneo cells. We found that both proliferation and migration in HTR-8/SVneo cells were significantly decreased compared to control cells treated with vehicle. Higher concentrations of l-1mT (2000 and 4000 μM) reduced HTR-8/SVneo cell proliferation, whereas lower concentrations (250−1000 μM) had no effect (Fig. 2D). At 1000 μM, l-1mT did suppress migration of HTR-8/SVneo cells, and this effect was greater at 2000 μM (Fig. 3A,B). Proliferation and migration were also suppressed in HTR-8/SVneo cells infected with shIDO1 and shIDO2 (Figs 2E and 3A,C). shIDO2, which is more efficient to suppress IDO expression compared to shIDO1, had a stronger inhibitory effect on both proliferation and migration.

### IDO knockdown inhibited STAT3 phosphorylation and MMP9 expression in trophoblast cells

**Figure 4:**
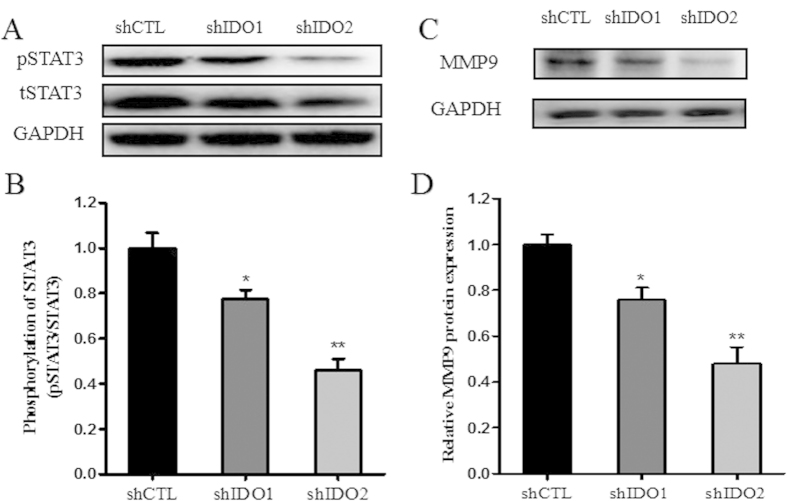
IDO knockdown suppressed STAT3 phosphorylation and MMP9 expression in HTR-8/SVneo cells. (**A,B**) Western blot analysis of pSTAT3 and MMP9 in HTR-8/SVneo cells infected with control or IDO-specific shRNA. GAPDH was used as an internal control. (**C,D**) Statistical analysis of western blotting results (n = 3; Student’s t-test; *p < 0.05, **p < 0.01).

To elucidate the mechanism by which IDO affects trophoblast proliferation and migration, we evaluated STAT3 signaling in trophoblast cells. We measured the expression of STAT3, phosphorylated STAT3 (pSTAT3) and their downstream target gene MMP9 by western blotting in IDO knockdown HTR-8/SVneo cells and control cells. The expression of pSTAT3 and MMP9 were significantly reduced in IDO knockdown HTR-8/SVneo cells compared with the control group (Fig. 4).

### Increased expression of STAT3 phosphorylation reversed the inhibitory effects of IDO knockdown on trophoblast cell proliferation and migration

**Figure 5:**
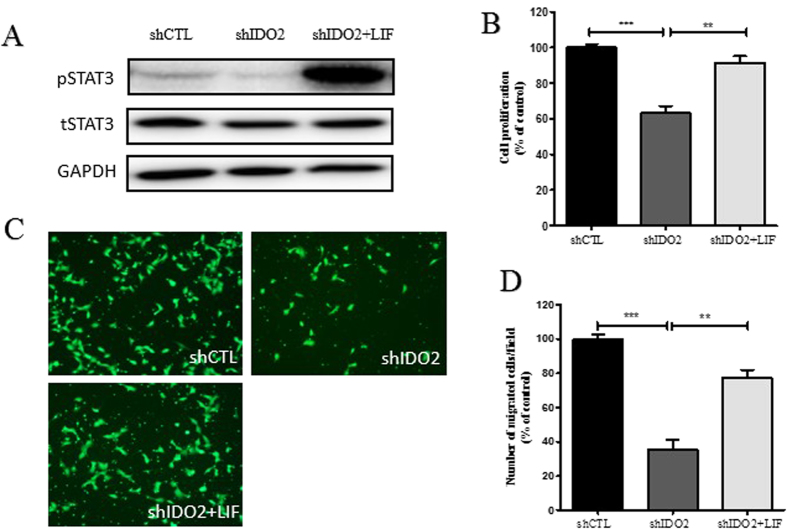
LIF partially reversed the inhibitory effects of IDO knockdown on trophoblast cell proliferation and migration. (**A**) After treatment with LIF, pSTAT3 expression in IDO knockdown HTR-8/SVneo cells was obviously increased. (**B**) LIF completely reversed the inhibitory effect on cell proliferation in IDO knockdown HTR-8/SVneo cells. (**C**,**D**) LIF partially reversed the inhibitory effect on migration in IDO knockdown HTR-8/SVneo cells (n = 3; Student’s t-test; **p < 0.01, ***p < 0.001).

We next sought to determine whether IDO regulates trophoblast cell proliferation and migration by affecting STAT3 signaling. To test this assumption, we treated IDO knockdown HTR-8/SVneo cells with the STAT3 phosphorylation activator LIF and conducted proliferation and migration assays. After treatment with LIF, pSTAT3 expression in IDO knockdown HTR-8/SVneo cells was significantly increased (Fig. 5A). In proliferation assay, LIF almost completely reversed the IDO knockdown-suppressed cell proliferation (Fig. 5B). LIF partly restored the migration ability of IDO knockdown in HTR-8/SVneo cells (Fig. 5C,D).

**Figure 6:**
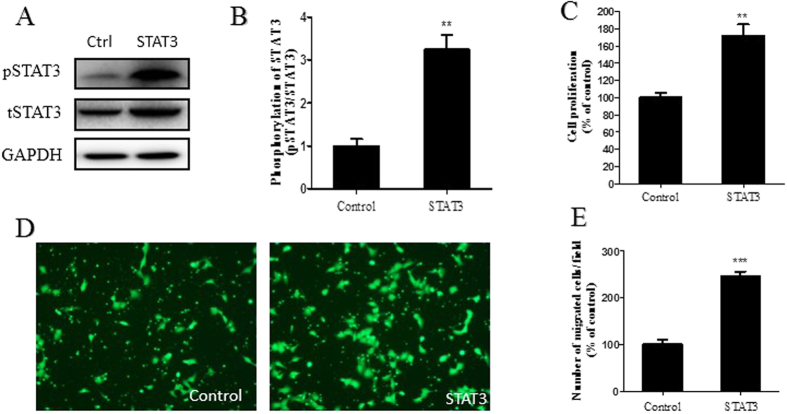
STAT3 overexpression reversed the inhibitory effects of IDO knockdown on trophoblast cell proliferation and migration. (**A**) Western blot analysis of pSTAT3 in STAT3 overexpressing shIDO2 HTR-8/SVneo cells. Total STAT3 was used as an internal control. (**B**) Statistical analysis of protein expression in A (n = 3; Student’s t-test; **p < 0.01). (**C**) Proliferation ability of STAT3-overexpressing shIDO2 HTR-8/SVneo cells was higher than the control group (n = 3; Student’s t-test; **p < 0.01). (**D,E**) Migration ability of STAT3-overexpressing shIDO2 HTR-8/SVneo cells was higher than the control group (n = 3; Student’s t-test; ***p < 0.001).

In addition, the lentivirus of STAT3 gene overexpression vector and control vector were infected into IDO knockdown HTR-8/SVneo (shIDO2 HTR8/SVneo) cells. Stably infected cells were used to perform proliferation and migration assays. The shIDO2 HTR8/SVneo cells with STAT3 gene overexpression exhibited significantly higher pSTAT3 expression than the control group (Fig. 6A,B). Cell proliferation and migration were also restored in the STAT3 gene overexpression group compared to the control group (Fig. 6C–E).

### IDO promotes cell proliferation and migration via the STAT3 signaling pathway

**Figure 7:**
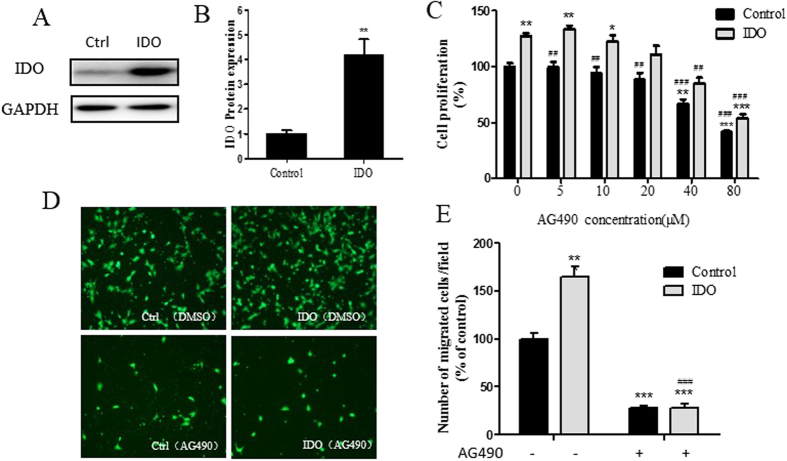
IDO promotes cell proliferation and migration via the STAT3 signaling pathway. (**A**) Western blot analysis of IDO in IDO-overexpressing HTR-8/SVneo cells. GAPDH was used as an internal control. (**B**) Statistical analysis of protein expression in A (n = 3; Student’s t-test; **p < 0.01). (**C**) Proliferation ability of IDO-overexpressing HTR-8/SVneo cells treated with vehicle or AG490 (n = 3; Student’s t-test; *p < 0.05, **p < 0.01, ***p < 0.001 in comparison to the control group treated with vehicle, ^##^p < 0.01, ^###^p < 0.001 in comparison to the IDO-overexpressing group treated with vehicle) (**D,E**) Migration ability of IDO-overexpressing HTR-8/SVneo cells treated with vehicle or AG490 (n = 3; Student’s t-test; **p < 0.01, ***p < 0.001 in comparison to the control group treated with vehicle, ^###^p < 0.001 in comparison to the IDO-overexpressing group treated with vehicle).

**Figure 8:**
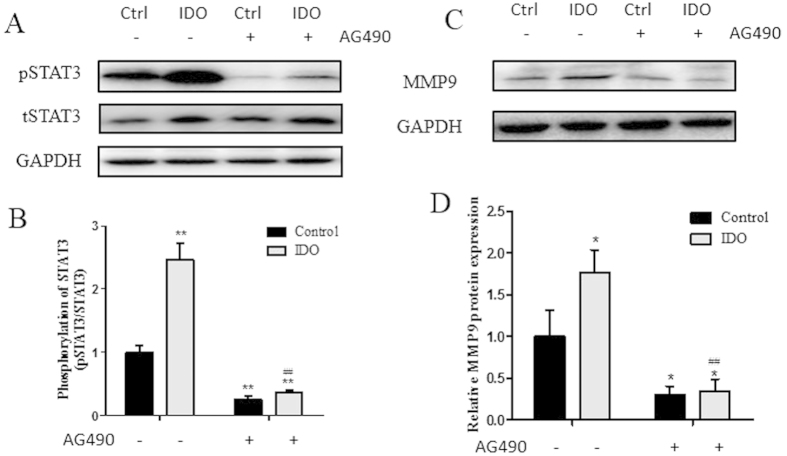
pSTAT3 and MMP9 expression in IDO-overexpressing HTR-8/SVneo cells. (**A**) Western blot analysis of pSTAT3 in IDO-overexpressing HTR-8/SVneo cells treated with vehicle or AG490. Total STAT3 was used as an internal control. (**B**) Statistical analysis of protein expression in A (n = 3; Student’s t-test; **p < 0.01 in comparison to the control group treated with vehicle, ^##^p < 0.01 in comparison to the IDO-overexpressing group treated with vehicle). (**C**) Western blot analysis of MMP9 in IDO-overexpressing HTR-8/SVneo cells treated with vehicle or AG490. GAPDH was used as an internal control (**D**) Statistical analysis of protein expression in A (n = 3; Student’s t-test; *p < 0.05 in comparison to the control group treated with vehicle, ^##^p < 0.01 in comparison to the IDO-overexpressing group treated with vehicle).

To further test whether IDO exerts its function through STAT3 signaling, we constructed an IDO gene overexpression HTR8/SVneo cell line and treated it with or without AG490. Then, proliferation and migration assays were carried out. IDO overexpression in HTR8/ SVneo cells showed significantly higher IDO protein expression compared to the control group (Fig. 7A,B). Meanwhile, the overexpression of IDO promoted cell proliferation and migration, and these facilitating effects could be abolished by the STAT3 signaling inhibitor AG490 (Fig. 7C–E). In addition, the overexpression of IDO upregulates pSTAT3 and MMP9 levels (Fig. 8). These results indicated that IDO promotes cell proliferation and migration via the STAT3 signaling pathway.

### Expression of phosphorylated STAT3 and MMP9 was decreased in URSA patients compared to normal pregnant women

**Figure 9:**
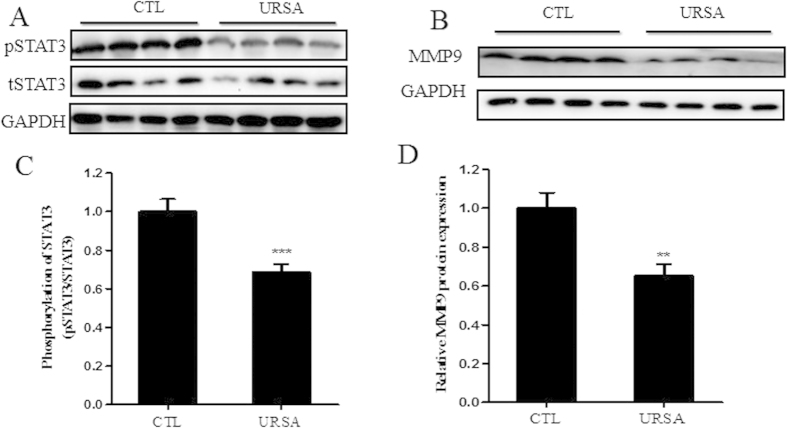
STAT3 phosphorylation and MMP9 expression were decreased in villi from URSA patients compared to those of normal pregnant women. (**A,B**) Western blot analysis of pSTAT3 and MMP9 expression in villi from normal pregnant women and URSA patients. GAPDH was used as an internal control. (**C,D**) Statistical analysis of western blotting results A and B (n = 20; Student’s t-test; **p < 0.01, ***p < 0.001).

We measured STAT3, pSTAT3 and MMP9 expression in the villi of URSA patients and normal pregnant women by western blotting. Villi from URSA patients had lower levels of phosphorylated STAT3 and MMP9 than villi from normal pregnant women (Fig. 9). These clinical findings were consistent with the results shown by IDO knockdown in cultured trophoblast cells. Taken together, these results indicate that IDO may regulate trophoblast proliferation and migration through the STAT3 signaling pathway.

## Discussion

During pregnancy, trophoblast proliferation, migration, invasion and spiral artery rebuilding are crucial processes for normal placental and fetal development. It has been known for decades that IDO is expressed in the placenta and important for the establishment and maintenance of pregnancy. There were high levels of IDO expression in the placenta and serum during pregnancy, however, IDO expression was reduced to the non-pregnancy level after delivery^6^. The use of the IDO inhibitor could result in abortion in pregnant mice. After IDO blockage, an inflammatory reaction was observed in the maternal-fetal interface^7^,^8^,^9^. In 1998, Munn *et al.* demonstrated that the fetus is protected from immune rejection by maternal T cells through a mechanism involving the IDO-dependent depletion of tryptophan^9^. IDO has been studied in various types of immune cells during pregnancy^12^,^20^,^21^,^22^,^23^,^24^. Trophoblast cells, the most important cells in the placenta in early pregnancy, have also been reported to express IDO^25^,^26^,^27^. However, the mechanisms connecting IDO and trophoblast function with pregnancy outcomes have not been investigated. Recent data suggest that IDO-positive tumor cells have a higher capacity for proliferation and metastasis than IDO-negative tumor cells^13^,^14^,^15^,^16^. As trophoblast cells are similar to tumor cells in some respects (e.g., proliferation and migration), IDO may play an important role in trophoblast function.

Previous reports have shown that the STAT3 signaling pathway plays an important role in regulating trophoblast cell growth, proliferation, migration and invasion^28^,^29^,^30^,^31^,^32^,^33^. Recent studies demonstrated that the inhibition of STAT3 phosphorylation resulted in the suppression of proliferation, migration, invasion and increased apoptosis of HTR-8/SVneo cells^31^,^32^,^33^. Certain cytokines and other molecules enhance HTR-8/SVneo cell invasion through STAT3 phosphorylation^34^,^35^,^36^.

In our study, we demonstrated that IDO protein expression and activity were decreased in villi of URSA patients compared to normal pregnant women, consistent with the findings of Ban *et al.*^10^. We further demonstrated that the inhibition and knockdown of IDO decreased the proliferative and migratory capacity of trophoblast cells, and IDO knockdown suppressed STAT3 phosphorylation and MMP9 expression. Increased expression of STAT3 phosphorylation reversed the inhibitory effects of IDO knockdown on trophoblast cell proliferation and migration to different degrees. The overexpression of IDO promotes cell proliferation and migration, and this facilitating effect could be abolished by the STAT3 signaling inhibitor AG490. Finally, we showed that STAT3 phosphorylation and MMP9 expression were decreased in women with URSA compared with normal pregnant women. It has been shown that the pattern of STAT3 expression from the first to the third trimester is highly similar to that of IDO^37^, and that STAT3 polymorphisms are linked to recurrent miscarriage^38^. Thus, it is likely that IDO insufficiency leads to impaired trophoblast cell proliferation and migration via the downregulation of STAT3 phosphorylation, eventually resulting in URSA. Our findings reveal new strategies in the regulation of trophoblast function and the way in which they may contribute to the pathogenesis of URSA.

## Additional Information

**How to cite this article**: Zong, S. *et al.* Dysregulated expression of IDO may cause unexplained recurrent spontaneous abortion through suppression of trophoblast cell proliferation and migration. *Sci. Rep.* **6**, 19916; doi: 10.1038/srep19916 (2016).
